# Living with a spouse or partner, material hardship, and mental distress among men and women during the COVID-19 pandemic in New York City

**DOI:** 10.3389/fpubh.2026.1794714

**Published:** 2026-05-14

**Authors:** Ao Shen

**Affiliations:** Department of Sociology, School of Social and Political Science, The University of Edinburgh, Edinburgh, United Kingdom

**Keywords:** COVID-19, inequality, mental distress, mental health, partner, poverty, spouse

## Abstract

**Background:**

The COVID-19 pandemic affected mental health worldwide. Mental health is considered related to marriage or cohabitation and material hardship. This study aims to explore the associations between living with a spouse or partner, material hardships, and mental distress among New York City residents during the pandemic. It focuses specifically on the relationship between living with a spouse/a spouse or partner and the risk of mental distress, as well as the role of material hardship in this association, examined separately for men and women.

**Methods:**

This study used cross-sectional data collected between late 2020 and early 2021 from the NYC Longitudinal Survey of Wellbeing. Chi-squared tests and several groups of logistic regression models were conducted.

**Results:**

Women and those who were not living with a spouse or partner were more likely to report material hardship, and mental distress than men and those living with a spouse or partner. Several material hardships were associated with a higher risk of mental distress. Among men, the results revealed that both living with a spouse and living with a spouse or partner were associated with a lower risk of mental distress, whereas material hardship moderated these relationships. For women, only living with a spouse or partner was found to be related to a lower risk of mental distress.

**Conclusion:**

The findings may reflect inequalities for some disadvantaged groups and provide implications for designing relevant social policies or delivering social services.

## 1 Introduction

The COVID-19 outbreak dramatically influenced daily life worldwide. In particular, the mental health of a large number of people suffered as a result of social isolation and adverse economic and health effects (1). During the pandemic, the general population reported increases in anxiety, depressive feelings, and suicidal thoughts (2). Simultaneously, it is possible that some relatively disadvantaged groups suffered from more financial difficulties, weaker social networks, and a high level of mental distress (3, 4).

Material hardship is defined as the lived experiences of economic deprivation, encompassing the inability to meet basic needs such as food, housing, utilities, and medical care due to financial constraints (5, 6). Previous research has illustrated a relationship between material hardship and poorer mental health, which has been observed in various groups, including low-income groups, those undergoing economic hardship (especially women), who are pregnant, or who have children (5, 7–10). Furthermore, multiple studies have demonstrated that those who experienced material or financial hardships were more likely to suffer from mental health problems during the COVID-19 pandemic (11–15). Thus, the following hypothesis is proposed:

H1: Material hardship is associated with a higher risk of mental distress.

Previous studies have confirmed a relationship between social support and improved mental health across different groups during the pandemic (4, 16–20). Spousal support is an important source of social support that may have played a more significant role during this difficult period. Several studies have examined the effects of spousal support on mental health. For example, Biehle and Mickelson (21) indicated that perceived spousal support was significantly related to a more positive mood and less anxiety and depression, whereas unperceived spousal support was only associated with less anxiety. Braithwaite and Holt-Lunstad (22) found a bidirectional positive association between romantic relationships and mental health, in which the causal arrow flowing from relationships to mental health was stronger. Regarding the influence of marriage or cohabitation on mental wellbeing during the pandemic, a twin analysis revealed greater life satisfaction and less depression among those who were married or cohabiting (23). Similarly, Jace and Makridis (24) observed better mental health outcomes among married respondents during the period. In a qualitative study on resilience during the pandemic, one participant said that romantic relationships greatly alleviated depression (25).

In addition, some research has examined whether marriage and cohabitation are differently associated with mental health. For example, Yucel and Latshaw (26), Amato (27), and Marcussen (28) reported that married individuals tend to have better mental health outcomes, including lower levels of depression, suicidal ideation, and alcohol use, than those who are cohabiting. However, other studies (29, 30) have found no significant differences in certain mental health outcomes between the two groups.

Given these mixed findings, it remains unclear whether distinguishing between marital status and broader partnership status is important for mental health outcomes. Living with a married spouse may reflect a more formal and potentially more stable relationship, whereas living with a spouse or partner captures a wider range of relationship types, including cohabitation. These differences may be related to variations in psychosocial conditions, coping resources, and relationship quality (28, 29). Therefore, this study distinguishes between individuals living with a married spouse and those living with a spouse or partner, and proposes separate hypotheses for these two groups.

Therefore, this study proposes the following hypotheses:

H2: Living with a spouse is associated with a lower risk of mental distress for men.
H3: Living with a spouse is associated with a lower risk of mental distress for women.

Meanwhile, in terms of the association between marriage or cohabitation and mental health, several studies have emphasized gender differences. For instance, Schwarzer and Gutierrez-Dona (31) discovered that spousal support was associated with a higher psychological quality of life for both men and women among 902 Costa Rican employees, but this relationship was stronger in men. Conversely, the relationship between spousal support and reduced depression was significant only in men. In a South Korean study, men reported higher levels of perceived spousal support than women in marriage (32). According to another recent study in New Zealand, the relationship between relationship status and perceived social support is stronger for men than women, which is the reason for the stronger connection between relationship status and wellbeing in men (33). However, Shin and Gyeong (34) argued that spousal support has a stronger protective effect against depression in women than in men.

So, the following hypotheses are proposed:

H4: Living with a spouse or partner is associated with a lower risk of mental distress for men.
H5: Living with a spouse or partner is associated with a lower risk of mental distress for women.

Furthermore, spousal relationships during the pandemic may have been negatively influenced by potential conflict and stress between couples (35). Although some studies have emphasized the positive impact of spousal support on mental health under negative conditions (23, 32, 36), others have noted that negative events, environments, or factors may harm marital relationships or reduce spousal support (37, 38). For instance, Neff and Karney (39) and Karney and Bradbury (40) found that more time and attention consumption, fewer sources of support, and a lack of energy and resources as a result of negative stressors were likely to undermine the marital wellbeing of low-income couples. Therefore, a possibility exists that material hardships may undermine the association between living with a spouse and improved mental health. According to research related to COVID-19 in terms of romantic relationships, factors related to COVID-19, including dramatic changes, external stressors, relationship conflicts, and individual vulnerabilities, exerted negative impacts on relationships between couples (41–44). Therefore, this study hypothesizes the following:

H6: Material hardship moderates the relationship between living with a spouse and mental distress for men.
H7: Material hardship moderates the relationship between living with a spouse and mental distress for women.
H8: Material hardship moderates the relationship between living with a spouse or partner and mental distress for men.
H9: Material hardship moderates the relationship between living with a spouse or partner and mental distress for women.

Previous research has made remarkable advances in examining the relationship between marriage or cohabitation and mental health, as well as changes in romantic/marital relationships in specific situations, including the COVID-19 pandemic. As a typical crisis scenario, the COVID-19 pandemic provides a suitable context for investigating the links between romantic/marital relationships, material hardships, and mental distress across different groups. However, although prior research has shown that women were more negatively influenced by the COVID-19 pandemic compared to men (3, 45), studies that consider gender differences regarding the association between romantic/marital relationships and mental health during the COVID-19 pandemic are relatively scarce. Therefore, this study aimed to explore the mechanisms of marriage or cohabitation, material hardship, and mental distress during the pandemic among men and women separately, which may provide a perspective on gender inequalities. Furthermore, considering that the COVID-19 pandemic has disproportionately affected some groups' mental health (45, 46), this study may also reflect potential inequalities among diverse cohorts and serve as a reference for promoting further changes.

This research aims to explore the links between living with a spouse or partner, material hardships, and mental distress among New York City residents during the pandemic. Specifically, it focuses on the relationship between living with a spouse/a spouse or partner and the risk of mental distress, as well as the role of material hardship in this association, examined respectively for men and women.

As an urbanized, diverse city with the largest population and highest population density in the United States, New York City experienced early surges in COVID-19 infections, such that the daily lives of New Yorkers were heavily influenced during the pandemic (47). This study conducted a cross-sectional analysis using secondary data from the New York City (NYC) Longitudinal Survey of Wellbeing to investigate the relationships among living with a spouse or partner, material hardship, and mental distress among New Yorkers during the pandemic.

## 2 Materials and methods

### 2.1 Data

This study used cross-sectional data analysis. The secondary dataset used was derived from the NYC Longitudinal Survey of Wellbeing (Poverty Tracker), which aims to provide a multidimensional and dynamic understanding of poverty, disadvantages, and wellbeing among New Yorkers (48). Launched in 2012, it collects detailed information on income poverty, material hardships, health problems, and other factors related to disadvantages from a representative sample of adults in NYC. For data collection, participants received the link to an online questionnaire via email and either completed it or opted for a phone interview to complete the surveys every 3 months.

Currently, Poverty Tracker has recruited and conducted surveys with five panels of participants, most of whom were recruited using random digit dialing across New York City. Given that each participant was associated with a unique phone number and tracked through a case management system across survey waves, the likelihood of the same individual appearing in multiple panels is expected to be very low. Data for the current study were collected between late 2020 and early 2021 during the pandemic, spanning the third (2017–2021) and fourth (2020–2024) collection panels. Because the primary lockdown period in New York City occurred between March 23 and August 16, 2020 (49), cross-sectional data collected closest to this period are likely to be more representative of the immediate conditions and impacts of the early phase of the COVID-19 pandemic.

Although all participants who completed the survey were asked the full set of questions relevant to this study, some declined to answer specific items or reported “don't know” or other uncertain responses. Consequently, sample sizes vary across analyses due to item-level non-response and missing data. The main sample for the logistic analysis was restricted to participants who completed all questions on whether they were living with a spouse, domestic partner, or neither, as well as their material hardships and mental health condition in related surveys, which included 1,498 participants (635 men, 863 women).

### 2.2 Measures

This study used three key measures: romantic relationship status, material hardship status, and mental health distress.

To examine romantic relationship status, this study employed two distinct dummy independent variables, each included in separate models to capture different aspects of living arrangements: one indicating whether the survey participant was living with a spouse, and another indicating whether the survey participant was living with either a spouse or a partner.

Living with a spouse: the survey participant was living with a married spouse.

Living with a spouse or partner: the survey participant was living with either a married spouse or a domestic partner (a live-in romantic partner).

To assess material hardships, the data encompassed five domains: food, housing, bills, general financial hardship, and medical care (50). This study used several measures of the material hardship statuses of the survey participants. These are dummy independent variables indicating whether the survey participant reported these kinds of hardships severely in the past 12 months. Specific information is listed as follows:

- Food hardship: running out of food or frequently worrying that food will run out without enough money to buy more.
- Housing hardship: having to stay in a shelter or another place not meant for regular housing or having to move in with others due to costs.
- Billing hardship: having utilities cut off due to the lack of money.
- Medical hardship: being unable to consult a medical professional due to cost.
- Financial hardship: frequently running out of money between paychecks or pay cycles.
- Severe hardship: the survey participants reported at least one of the abovementioned forms of hardship in the past 12 months.

The six-item Kessler Psychological Distress Scale (K6) was used to measure mental health distress in survey participants (51). This scale has one dimension with six items (e.g., *During the past 30 days, about how often did you feel restless or fidgety?*) The items were rated on a five-point scale ranging from 0 (*None of the time*) to 4 (*All of the time*). The total scores ranged from 0 to 24, with high scores indicating high levels of mental distress. Low, moderate, and severe mental distress were represented by total scores ranging from 0 to 7, 8 to 12, and 13 to 24, respectively. Survey participants with a total score of 8 or higher were classified as reporting mental distress. This study used the following dummy independent variables to indicate whether a survey participant reported each of the following statuses:

- Moderate mental distress: total scores ranging from 8 to 12.
- Severe mental distress: total scores ranging from 13 to 24.
- Mental distress: total scores ranging from 8 to 24.

The control variables included age, race, education level, borough, employment status, income, poverty status, presence and number of children, immigrant status, medical insurance status, and health status.

### 2.3 Data analysis plan

To measure the relationship between living with a spouse or partner, material hardship, and mental distress among adults in New York City during the pandemic, this study conducted chi-squared tests and several groups of logistic regression models. All logistic regression analyses were conducted among male and female survey participants.

Table 1 summarizes the percentages of male and female survey participants living with a spouse, living with a partner, and reporting financial, food, housing, billing, medical, financial, or severe hardship (defined as reporting at least one of the aforementioned forms of hardship in the past 12 months), as well as reporting moderate and severe mental distress. Chi-squared tests were used to detect significant differences between men and women.

**Table 1:** Percentage of male and female adults in New York who were living with a married spouse or cohabiting partner, and the percentage who reported various types of material hardship or mental distress during the pandemic.

Variable	Men	Women
Living with a spouse^***^	44.40% (*n* = 1,124)	34.41% (*n* = 1,607)
Living with a spouse or partner^***^	52.94% (*n* = 1,124)	39.64% (*n* = 1,607)
Food hardship^*^	6.21% (*n* = 1,127)	8.75% (*n* = 1,612)
Housing hardship	6.03% (*n* = 1,127)	6.08% (*n* = 1,612)
Billing hardship	6.57% (*n* = 1,127)	7.75% (*n* = 1,612)
Medical hardship	15.79% (*n* = 1,127)	18.24% (*n* = 1,612)
Financial hardship^**^	9.05% (*n* = 1,127)	12.41% (*n* = 1,612)
Severe hardship^***^	25.91% (*n* = 1,127)	33.31% (*n* = 1,612)
Moderate mental distress	37.95% (*n* = 983)	40.66% (*n* = 1,414)
Severe mental distress^**^	8.85% (*n* = 983)	12.59% (*n* = 1,414)

Chi-squared tests were used to detect significant differences between men and women.

^*^p < 0.05, ^**^p < 0.01, ^***^p < 0.001.

In Tables 2, 3, male and female survey participants are classified according to whether they were living with a spouse/a spouse or partner; the percentages of the subgroups who reported the abovementioned material hardship and mental distress are displayed. Chi-squared tests were also used to detect significant differences between the subgroups who were and were not living with a spouse/a spouse or partner.

**Table 2:** Percentage of male and female New Yorkers who were/were not living with a married spouse and who reported various types of material hardship or mental distress during the pandemic.

Variable	Men		Women	
	Living with a spouse	Not living with a spouse	Living with a spouse	Not living with a spouse
Food hardship	4.21% (*n* = 499)	7.84%^*^ (*n* = 625)	3.44% (*n* = 553)	11.57%^***^ (*n* = 1,054)
Housing hardship	2.40% (*n* = 499)	8.80%^***^ (*n* = 625)	4.16% (*n* = 553)	7.12%^*^ (*n* = 1,054)
Billing hardship	3.21% (*n* = 499)	9.28%^***^ (*n* = 625)	3.25% (*n* = 553)	10.15%^***^ (*n* = 1,054)
Medical hardship	11.42% (*n* = 499)	19.20%^***^ (*n* = 625)	15.01% (*n* = 553)	19.82%^*^ (*n* = 1,054)
Financial hardship	7.41% (*n* = 499)	10.24% (*n* = 625)	7.41% (*n* = 553)	14.99%^***^ (*n* = 1,054)
Severe hardship	18.43% (*n* = 499)	31.84%^***^ (*n* = 625)	25.14% (*n* = 553)	37.57%^***^ (*n* = 1,054)
Moderate mental distress	35.35% (*n* = 430)	40.00% (*n* = 550)	36.76% (*n* = 487)	42.80%^*^ (*n* = 923)
Severe mental distress	3.95% (*n* = 430)	12.55%^***^ (*n* = 550)	8.42% (*n* = 487)	14.63%^**^ (*n* = 923)

The “Not Living with a Spouse” groups comprise individuals who were not living with a romantic partner, as well as those cohabiting with an unmarried partner.

Chi-squared tests were used to detect significant differences between the men and women.

^*^p < 0.05, ^**^p < 0.01, ^***^p < 0.001.

**Table 3:** Percentage of male and female New Yorkers who were/were not living with a married spouse or cohabiting partner and who reported various types of material hardship or mental distress during the pandemic.

Variable	Men		Women	
	Living with a spouse/partner	Not living with a spouse/partner	Living with a spouse/partner	Not living with a spouse/partner
Food hardship	5.04% (*n* = 595)	7.56% (*n* = 529)	4.08% (*n* = 637)	11.86%^***^ (*n* = 970)
Housing hardship	4.20% (*n* = 595)	7.94%^**^ (*n* = 529)	4.40% (*n* = 637)	7.22%^*^ (*n* = 970)
Billing hardship	5.04% (*n* = 595)	8.32%^*^ (*n* = 529)	4.55% (*n* = 637)	9.90%^***^ (*n* = 970)
Medical hardship	13.78% (*n* = 595)	17.96% (*n* = 529)	16.33% (*n* = 637)	19.38% (*n* = 970)
Financial hardship	7.56% (*n* = 595)	10.59% (*n* = 529)	8.63% (*n* = 637)	14.85%^***^ (*n* = 970)
Severe hardship	22.35% (*n* = 595)	29.87%^**^ (*n* = 529)	27.79% (*n* = 637)	36.91%^***^ (*n* = 970)
Moderate mental distress	36.19% (*n* = 514)	39.91% (*n* = 466)	38.06% (*n* = 557)	42.44% (*n* = 853)
Severe mental distress	6.03% (*n* = 514)	11.80%^**^ (*n* = 466)	8.80% (*n* = 557)	14.89%^**^ (*n* = 853)

Chi-squared tests were used to detect significant differences between the men and women.

^*^p < 0.05, ^**^p < 0.01, ^***^p < 0.001.

In the first and second groups of logistic regression models, the key independent variables were whether the survey participant was living with a spouse/a spouse or partner and reported food, housing, billing, medical, or financial hardships. The dependent variable was whether the survey participant reported mental distress (total K6 scores ranged from 8 to 24), while the control independent variables were used.

**Figure 1:**
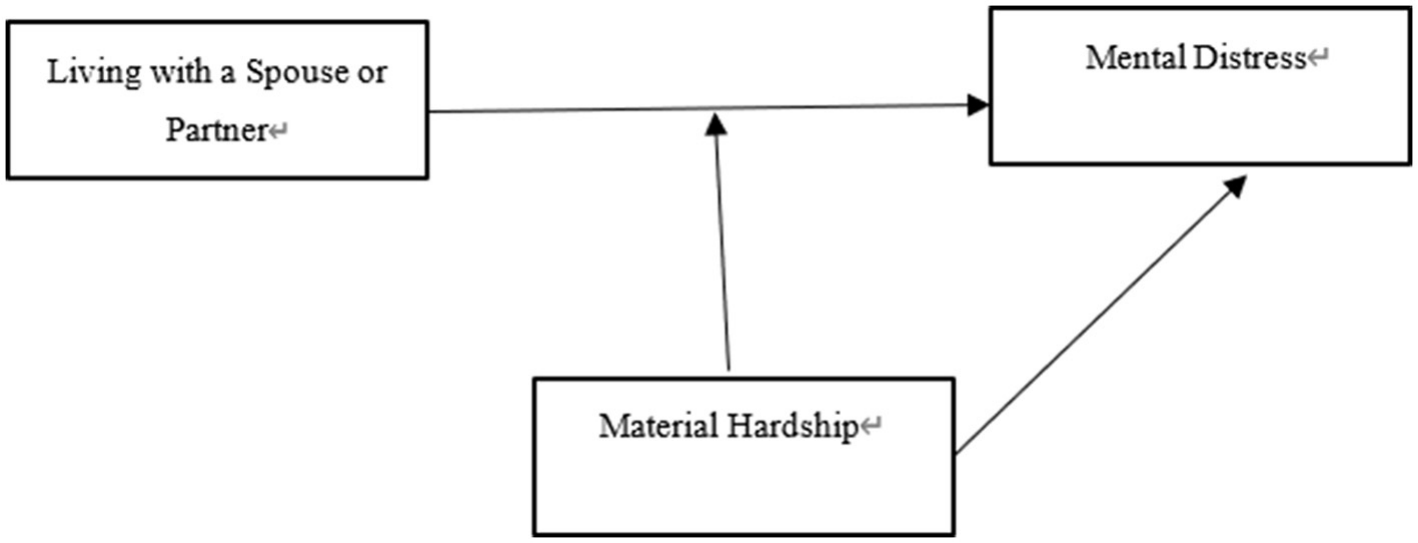
Planned model.

Given the potential effect of material hardship as a moderator, the study used only living with a spouse/a spouse or partner and reporting severe hardship as the key independent variables and added their interaction term in the third and fourth groups of logistic regression models; however, the dependent variable was the same as in the two previous models. Figure 1 depicts the hypothesized relationships between the main variables in the two regression groups. The remaining regression models were used to further prove the moderating effects of severe hardship.

## 3 Results

Table 1 presents the percentages of male and female New Yorkers who were living with a spouse or partner, and the percentage of those who reported various types of material hardship or mental distress during the pandemic. According to the results, in NYC, a higher percentage of men were living with a spouse, and a higher percentage of men were living with a partner, while a lower percentage of men reported material hardship or mental distress.

Regarding marriage and cohabitation, the relationship between gender and living with a spouse or partner was statistically significant. In terms of material hardship, the relationship between gender and reporting at least one type of hardship was statistically significant at the 0.001 level. Specifically, the relationships among gender, food, and financial hardship were found to be statistically significant. For mental distress, the association between gender and severe mental distress was statistically significant at the 0.01 level.

Tables 2, 3 show the percentages of male and female New Yorkers who did or did not report various types of material hardships or mental distress while living with a spouse/spouse or partner during the pandemic. According to the results, a higher percentage of men and women who did not live with a spouse or partner reported material hardship or mental distress in NYC than those who did.

Among male New Yorkers, the relationship between living with a spouse and reporting at least one type of hardship or severe mental distress was statistically significant at the 0.001 level, while the association between living with a spouse or partner and reporting at least one type of hardship or severe mental distress was statistically significant at the 0.01 level. Regarding female New Yorkers, the relationship between living with a spouse/a spouse or partner and reporting at least one type of hardship was statistically significant at the 0.001 level, while the association between living with a spouse/a spouse or partner and reporting severe mental distress was statistically significant at the 0.01 level.

Table 4 shows the results of the two logistic regressions that aimed to predict the relationships between mental distress and living with a spouse/spouse or partner and reporting each type of material hardship among males and females separately.

**Table 4:** Odds ratio and 95% confidence interval of living with a married spouse/a married spouse or cohabiting partner and reporting various types of material hardship in mental distress.

Variable	Model 1 (Men)	Model 1 (Women)	Model 2 (Men)	Model 2 (Women)
Living with a spouse	0.455^**^	0.716		
	(0.286, 0.724)	(0.502, 1.021)		
Living with a spouse or partner			0.599^*^	0.656^*^
			(0.389, 0.922)	(0.462, 0.931)
Food hardship	1.213	2.852^**^	1.195	2.765^**^
	(0.505, 2.914)	(1.344, 6.054)	(0.498, 2.865)	(1.300, 5.881)
Housing hardship	2.283	2.697^**^	2.376	2.598^**^
	(0.902, 5.775)	(1.322, 5.502)	(0.944, 5.978)	(1.271, 5.309)
Billing hardship	1.224	1.107	1.388	1.115
	(0.530, 2.827)	(0.549, 2.234)	(0.599, 3.215)	(0.552, 2.253)
Medical hardship	3.411^***^	1.751^**^	3.541^***^	1.780^**^
	(1.976, 5.890)	(1.152, 2.662)	(2.052, 6.110)	(1.170, 2.709)
Financial hardship	2.191^*^	2.996^***^	2.094	3.062^***^
	(1.018, 4.716)	(1.679, 5.347)	(0.976, 4.491)	(1.713, 5.475)
Observations	635	863	635	863
Pseudo R-squared value	0.1662	0.1378	0.1596	0.1396

^*^p < 0.05, ^**^p < 0.01, ^***^p < 0.001.

According to the regression results for men, holding all other independent variables constant, male New Yorkers living with a spouse/a spouse or partner had 54.5% lower/40.1% lower odds of reporting mental distress, compared to those who were not. In other words, the odds of reporting mental distress among those living with a spouse/a spouse or partner were approximately 0.455/0.599 times those of their counterparts, indicating a substantially lower likelihood of reporting mental distress among this group. Therefore, these findings indicate that a relationship exists between living with a spouse/a spouse or partner and a lower risk of mental distress among male New Yorkers, with the former being stronger and statistically more robust. In addition, medical hardship was significantly associated with a higher risk of mental distress among male New Yorkers.

For women, holding other predictor variables constant, female New Yorkers living with a spouse or partner had 34.4% lower odds of reporting mental distress compared to those who were not. Stated differently, the odds of reporting mental distress among those living with a spouse or partner were approximately 0.656 times those of their counterparts, demonstrating a notably lower likelihood of reporting mental distress among this group. These results indicate an association between living with a spouse or partner and a lower risk of mental distress among women in New York City. However, living with a spouse was not significantly associated with reporting mental distress. Associations between a higher risk of mental distress and food, housing, medical, and financial hardships were also found to be significant among women in New York during the pandemic. These findings support H2, H4, and H5 but not H3.

Table 5 presents the results of the regressions when considering reporting at least one type of material hardship as a moderator of the relationship between living with a spouse/a spouse or partner and mental distress; thus, the study added an interaction term.

**Table 5:** Odds ratio and 95% confidence interval of living with a married spouse/a married spouse or cohabiting partner and reporting at least one types of material hardship in mental distress.

Variable	Model 3 (Men)	Model 3 (Women)	Model 4 (Men)	Model 4 (Women)
Living with a spouse	0.378^***^	0.732		
	(0.226, 0.632)	(0.490, 1.093)		
Living with a spouse or partner			0.474^**^	0.641^*^
			(0.289, 0.775)	(0.429, 0.956)
Severe hardship	3.045^***^	2.896^***^	2.904^***^	2.820^***^
	(1.775, 5.224)	(1.907, 4.398)	(1.614, 5.224)	(1.821, 4.366)
Living with a married spouse × severe hardship	2.562^*^	0.808		
	(1.066, 6.155)	(0.418, 1.559)		
Living with a spouse or partner × severe hardship			2.486^*^	0.898
			(1.087, 5.686)	(0.474, 1.702)
Observations	635	863	635	863
Pseudo R-squared	0.1771	0.1199	0.1716	0.1222

^*^p < 0.05, ^**^p < 0.01, ^***^p < 0.001.

Table 6 illustrates the results of the regressions of the relationships between mental distress and living with a spouse/a spouse or partner for men in New York with or without at least one type of material hardship during the pandemic.

**Table 6:** Odds ratio and 95% confidence interval of living with a married spouse/a married spouse or cohabiting partner in reporting mental distress among men.

Variable	Model 5 (with hardship)	Model 5 (no hardship)	Model 6 (with hardship)	Model 4 (no hardship)
Living with a spouse	1.327	0.408^**^		
	(0.493, 3.570)	(0.232, 0.718)		
Living with a spouse or partner			1.183	0.521^*^
			(0.504, 2.778)	(0.303, 0.896)
Observations	177	458	177	458
Pseudo R-squared value	0.1249	0.1483	0.1241	0.1413

^*^p < 0.05, ^**^p < 0.01.

According to the findings, the models, particularly those for the moderating effect of severe hardship, were only valid for men in New York during the pandemic. Thus, H1, H6, and H8 are supported but not H7 and H9.

Based on these results, this study observed statistically significant associations between self-reported mental distress and living with a spouse/a spouse or partner among men in New York who did not report material hardship during the pandemic. Thus, material hardship had a moderating effect on the relationship between living with a spouse or partner and mental distress among men living in NYC.

## 4 Discussion

This study explored the relationship between living with a spouse/a spouse or partner, material hardships, and mental distress among male and female adults in New York during the COVID-19 pandemic. The following conclusions were drawn.

First, reporting material hardships, especially medical hardships, was related to self-reported mental distress among New Yorkers during the pandemic, which is consistent with the results of previous studies (9, 12–15).

Compared with male New Yorkers, women may have been more vulnerable during the pandemic because of the relationship between being female and a higher risk of at least one type of hardship and severe mental distress, as well as the relationship between reporting certain types of material hardship and a higher risk of mental distress among women only. At the same time, compared with adults in New York who were living with a spouse/a spouse or partner, those who were not may have been more vulnerable during the pandemic because of the associations between living with a spouse/a spouse or partner and a lower risk of both material hardship and mental distress among men and women.

Furthermore, the study revealed several findings among male New Yorkers regarding the relationships between living with a spouse, living with a spouse or partner, and mental distress. Specifically, the study found that living with a spouse or partner was related to a lower risk of mental distress, which is in line with the results of earlier research demonstrating that marriage or cohabitation is correlated with improved mental health during the pandemic (23, 24). The study also identified that the association between living with a spouse and a lower risk of mental distress was stronger than that between living with a spouse or partner and a lower risk of mental distress among men. This is consistent with the results of previous studies that reported that marriage is more beneficial than cohabitation (23, 26–28). Furthermore, reporting at least one type of material hardship moderates the relationship between living with a spouse/a spouse or partner and mental distress. Therefore, according to other studies that indicated that adverse external factors negatively influenced couple relationships during the COVID-19 pandemic (41–44), the effect of material hardship may have played a negative role in the influence of potential spousal support on mental distress.

However, for female New Yorkers, the study observed an association only between living with a spouse or partner and a lower risk of mental distress. Living with a spouse was found to be related to a lower risk of mental distress among men, but not women. Moreover, the association between living with a spouse or partner and a lower risk of mental distress was stronger among men than women. Given the differences in the findings for male and female New Yorkers, men may mentally benefit more from romantic relationships than women, which is in accordance with the results of other related studies (31–33).

A potential explanation for some of the findings is the division of labor within couples. Research on housework during the pandemic revealed that women not only bore a disproportionately greater burden of household tasks compared with men, but also experienced a significant increase in this burden relative to pre-pandemic levels (52–54). Additionally, previous studies have shown that married women, but not men, spend more time on housework compared to those in cohabiting relationships, where the division of household labor tends to be more egalitarian than in marriages (55–58). Therefore, it is likely that married women undertook more housework than both their cohabiting counterparts and married men during the pandemic. This may explain why living with a spouse or partner was associated with a lower risk of mental distress among women, whereas this association was not observed for women living solely with a spouse, but was present among men living with a spouse.

Furthermore, prior research has indicated that, within couples, men tend to be more reactive to economic hardship than women (59, 60). This may account for why the moderating effect of material hardship on the relationship between living with a spouse or partner and a lower risk of mental distress was observed only among men, and not women.

This study has several limitations. First, the study was unable to quantify the level of support from a spouse or partner, which could permit a more accurate examination of the impact of romantic relationships on mental wellbeing. Second, this study employed a cross-sectional design using data collected between late 2020 and early 2021, thus capturing only the early phase of the COVID-19 pandemic. This timeframe was selected because it represents a critical period in New York City, characterized by widespread lockdown measures and heightened social and economic disruptions (49). However, the pandemic continued beyond this period, and limiting the analysis to the first year may not fully capture changes in social conditions, coping mechanisms, or mental health outcomes in later stages. Future research could incorporate datasets covering subsequent phases of the pandemic to provide a more comprehensive understanding of these dynamics. Additionally, longitudinal or panel data designs would enable comparisons across pre-pandemic, pandemic, and post-pandemic periods, thereby offering further insights into the specific impact of the pandemic on the relationship between cohabitation with a spouse or partner and mental distress. Lastly, studies incorporating more precise measures of spousal support may further explore the mechanisms underlying the effects of spousal support and material hardship on mental health.

In addition, although the material hardship measures were intended to capture a broad range of experiences, some variables may conceptually overlap to a certain extent, which should be considered when interpreting the results. For example, financial hardship reflects a general indicator of economic strain, whereas other measures capture domain-specific hardships such as difficulties affording food, housing, bills, or medical care. In practice, these experiences may be closely related, as financial strain can manifest through these specific domains.

Another important limitation concerns the interpretation of the observed associations. While the statistical analyses employed in this study identify relationships between cohabitation with a spouse or partner and lower mental distress, the cross-sectional design does not allow for causal inference. Specifically, it is not possible to determine whether cohabitation exerts a protective effect on mental health, or whether individuals with better mental health are more likely to enter into or remain in cohabiting arrangements. This potential selection mechanism represents an important alternative explanation of the findings and should be considered when interpreting the results.

Furthermore, although this research pointed out the links between living with a spouse/partner, material hardship, and mental distress as well as some differences among various groups, it did not delve more deeply into the underlying factors, especially the inequalities leading to them. For example, social reproduction and the division of labor are potential contributors to these differences. Therefore, examining these factors and their impact on potential inequalities is a direction for future studies.

Finally, this study only focused on the residents of New York City during the pandemic, which limits the generalization of the findings.

In terms of practical applications, this research is likely to provide useful information for developing relevant social policies or delivering better social services for disadvantaged groups, particularly during these difficult periods. Some findings, including the relationship between being female and a higher risk of material hardship, or suffering from mental distress, indicate the adverse situations of some relatively disadvantaged groups and potential inequalities. Therefore, people who experience material hardship (especially medical hardship), particularly women or those who do not live with a spouse or partner, are more prone to mental distress. Consequently, their mental health is more likely to be adversely affected. Therefore, to better support those who are relatively vulnerable and address inequalities, it is rational to consider how to increase their accessibility to mental health care, mitigate their material hardships, and improve related preventive interventions, such as strengthening support from their families and communities.

## Data Availability

The data analyzed in this study is subject to the following licenses/restrictions: the datasets presented in this article are not readily available because they are subject to data access restrictions and data governance policies. Requests to access these datasets should be directed to Center on Poverty and Social Policy at the Columbia University School of Social Work, cpsp@columbia.edu.

## Author's note

The data used in this study was obtained from the New York City (NYC) Longitudinal Survey of Wellbeing, which was funded by the Robin Hood Foundation.
